# The Role of Adsorption in Agarose Gel Cleaning of Artworks on Paper

**DOI:** 10.3390/gels11120965

**Published:** 2025-11-29

**Authors:** Teresa T. Duncan, Michelle R. Sullivan, Amy Elizabeth Hughes, Kathryn M. Morales, Edwin P. Chan, Barbara H. Berrie

**Affiliations:** 1 Scientific Research, National Gallery of Art, 2000 South Club Drive, Landover, MD 20785, USA; k-morales@nga.gov; 2 Department of Paper Conservation, J. Paul Getty Museum, 1200 Getty Center Drive, Los Angeles, CA 90049, USA; msullivan@getty.edu; 3 Paper Conservation, National Gallery of Art, 2000 South Club Drive, Landover, MD 20785, USA; ahughes@loc.gov; 4 Materials Science and Engineering Division, National Institute of Standards and Technology, Gaithersburg, MD 20899, USA; edwin.chan@nist.gov; 5 Independent Researcher, Washington, DC 20010, USA

**Keywords:** agarose, hydrogel, paper conservation, cleaning, adsorption, tidelines

## Abstract

We present an exploration of an overlooked process in gel cleaning that promotes efficient cleaning of discoloration and stains from artworks on paper: adsorption. Agarose, in both solid and gelled forms, is an efficient adsorbent of crystal violet, which is used here as a marker to assess the capability of a system to immobilize solutes. Incorporating additional adsorbents, either 1% by mass microcellulose or silica gel, into the gel before casting greatly improves the efficiency of removing and retaining dye from water. This addition induces a slight (2×) increase in the elastic modulus but results in no impactful change in the handling properties for conservation practice. We show that the addition of silica gel increases the efficacy of removing water-soluble degradation products from a sheet of historic book paper. A case study of a water-damaged eighteenth-century print, with element maps collected using mapping µX-ray fluorescence analysis before and after gel cleaning, demonstrates that microcellulose-containing gels can be used to remove water-soluble salts from the print. This work provides a new methodology for tailoring gels to target specific conservation treatment outcomes. Specifically, efficient adsorption of solubilized material increases the efficacy of the gel cleaning and minimizes redeposition.

## 1. Introduction

The removal of stains and degradation products from artworks on paper can improve the appearance and increase the longevity of an artwork. The cleaning of artworks on paper requires careful selection of cleaning methods best suited to the properties of the stain, the artwork, and the desired outcome. Here, we present a phenomenological study of the role of adsorption in gel cleaning. Specifically, this paper shows that agarose gels can adsorb stain molecules and that adding additional adsorbents increases solubilization of material and prevents redeposition.

Paper composition varies but generally consists of cellulosic fibers with additives such as fillers, sizing, coatings, and colorants that together form a complex, composite network material [1]. Although cellulose can be a highly stable material in a controlled environment, it is susceptible to chemical degradation, leading to discoloration and embrittlement. While chemical degradation occurs naturally, it is exacerbated by external factors (e.g., contact with acidic mounts, light exposure, elevated temperature) or specific incidents (e.g., water damage) [2]. During conservation treatment, immersing paper in water allows for the solubilization, and thereby removal, of water-soluble degradation products produced from hydrolysis, oxidation, cross-linking, microorganisms, pollution, and more [3]. The intended result for an artwork undergoing treatment is a decrease in the discoloration (typically yellowness) of the degraded paper support and an increase in the pliancy and chemical stability of the paper due to the removal of acidic compounds. The benefits of washing must be balanced with potential drawbacks, which could include, for example, a loss of sizing or a decrease in strength [4]. This consideration is one of many for the conservator: how can the benefits be maximized and the risks be minimized for a selected treatment method?

In an effort to minimize certain risks involved with aqueous treatments, conservators and conservation scientists have explored the use of hydrogels for cleaning paper and other types of art substrates [5,6]. Highly retentive gels limit the amount of water penetration into paper, thereby minimizing swelling of fibers [7]. Gel residues, resulting from the physical transfer of gel material to the work of art, are minimized but not always entirely prevented by using a rigid gel system [8,9,10]. Strategies to reduce the risk of residues remaining after treatment can be employed, including the use of a barrier tissue (which can decrease efficacy) [9] and ensuring that any applied pressure (via placement of a weight) is sufficient for efficient contact but does not exceed forces that can induce fracture within the gel [11]. Different gelators with tailored chemical functionalization, such as methylation of gellan gum [12], or innovative morphologies, such as microgel structures [13], increase efficacy and decrease cleaning time.

The general procedure for using a hydrogel for cleaning paper involves placing a gel, either overall or in a localized area, in contact with either dry or lightly dampened paper. Ideally, water permeates into the paper, stains (from internal or external origins) are solubilized, and the stain components diffuse into the hydrogel and are held within it. Solubilized stain components that migrate within the paper, rather than to the hydrogel, appear as tidelines in localized treatments. Control over water delivery is critical for avoiding the formation of tidelines. Conservators control gel treatments through variations in polymer concentration, casting thickness, dwell time, addition of cross-linking agents, and other means.

**Figure 1:**
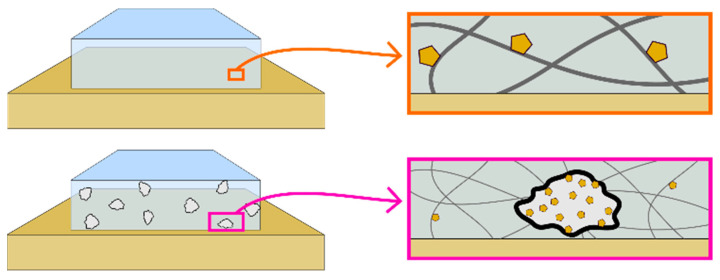
Schematic illustrating the adsorption of solubilized materials (yellow pentagons) to either the solid-like agarose network (**top**) or the surface of added adsorbents (**bottom**) after absorption of solubilized material into the bulk of the gel.

Here, we propose that the process of solute adsorption in a gel is a critical aspect of effective gel cleaning in art conservation. Gels made with higher concentrations of agarose are more effective in the uptake of water-soluble degradation products from paper [14]. We show that, upon absorption of solubilized material into a gel, solid and gelled agarose acts like an adsorbent. Adsorption is the process of molecules in a gas or liquid state (here, solubilized in the water) adhering to the surface of a solid (here, the solid or solid-like agarose network). Adsorption, whether of physical and/or chemical (also referred to as ‘chemisorption’) origin, of solubilized material in the water phase of the gel decreases redeposition from solution onto the surface being cleaned, and this increases cleaning efficacy. In general, the development of new materials requires an understanding of the processes in play so that modifications can target specific outcomes. To this end, we incorporate additional adsorbents, such as microcellulose (MC) and silica gel (SG), into the agarose gel to boost the cleaning efficacy of the material. The schematic presented in Figure 1 illustrates this process of adsorption to either the gel network (top) or additional adsorbent (bottom) that is mixed into the gel during preparation. We show how a more thorough understanding of agarose cleaning allows for simple and effective improvements to gels for conservation. This research serves as an initial exploration of the effects of added adsorbents within an agarose gel network; further work is needed to analytically assess the interactions between solubilized materials, different gel materials, and other types of additives to allow for broader implementation of these findings into conservation practice.

## 2. Results and Discussion

### 2.1. Adsorption of Crystal Violet Dye

The ability of agarose, both in solid (as received) and gelled form, to act as an adsorbent was assessed by adding the solid or gelled agarose to a crystal violet (CV) solution. CV is employed here as a marker of adsorption for two reasons: (1) it is highly soluble in water, making its removal from water a challenge, and (2) its intense purple color allows easy visualization of the adsorption process and measurement with UV–Vis spectroscopy. The capability of solid agarose to act as an adsorbent is described in the water treatment literature. Solid agarose, in dried cryogel or xerogel form to increase porosity and surface area, can purify dyes in water basins, as water sources can become contaminated with dyes such as CV or methylene blue from various industrial processes. Specifically, freeze-dried agarose gels can act as an adsorbent to remove methylene blue from wastewater [15]. Agarose cryogels and aerogels, subjected to either freeze drying or supercritical drying, respectively, are efficient adsorbents of CV [16]. But the question for art conservation practice remained: do hydrated agarose gels also act as adsorbents? To address this question, we studied the uptake of CV from solution by solid and gelled (hydrated) agarose.

**Figure 2:**
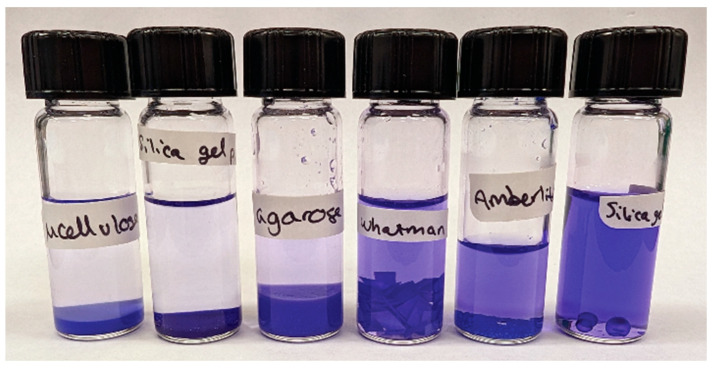
An aqueous 3 × 10^−5^ M solution of crystal violet was added to vials containing 50 mg solid compounds. The mixture was shaken, and the photograph was collected one hour later. Left to right, the vials contain: MC, SG, solid agarose, Whatman filter paper, Amberlite XAD7HP, and silica gel desiccant beads. The vials are arranged left to right from most to least effective for crystal violet removal from solution. Results from absorption measurements can be found in Table 1.

To monitor the adsorption capacity of solid agarose, an aqueous solution of 3 × 10^−5^ M CV was added to a vial containing solid agarose, and the mixture was shaken. Upon mixing, a decrease in color intensity of the solution was noted immediately, indicative of rapid adsorption of CV onto the solid agarose surface. The decrease in the concentration of CV in solution can be noted visually in Figure 2. In relation to other tested compounds, agarose was less efficient than the MC and SG, both of which are known to be efficient adsorbents with fine particle size (approximately 20 µm length and 50 µm diameter, respectively, with the SG used for column chromatography). Agarose was more effective than Whatman filter paper, Amberlite XAD7HP, and silica gel beads (of approximately 3 mm diameter, commonly used as a desiccant). The efficacy of CV removal from solution by the various materials was measured after 1 h with UV–Vis spectroscopy from the decrease in the CV absorbance maximum, with results tabulated in Table 1. Absorption measurements indicate that the agarose removed 90% of the CV from solution, and prior studies suggest this occurs via electrostatic interactions such as hydrogen bonding with the surface of the solid agarose [16,17]. The only compounds that were found to be more effective than solid agarose after an hour were MC and SG, which removed 93% and 94%, respectively. This experiment showed that solid agarose is an effective adsorbent for CV. No appreciable swelling of agarose was noted visually at room temperature, but gelators that do swell in water at room temperature (e.g., gellan) may exhibit different behavior. Importantly, this adsorption onto the agarose surface takes place rapidly, an important consideration for conservation practice. Fast adsorption helps minimize the redeposition of the absorbed stain materials into the artwork undergoing conservation treatment. Amberlite and the silica gel desiccant were highly effective over days, but too slow to be useful during most paper conservation gel treatments, which typically involve contact times under an hour.

**Figure 3:**
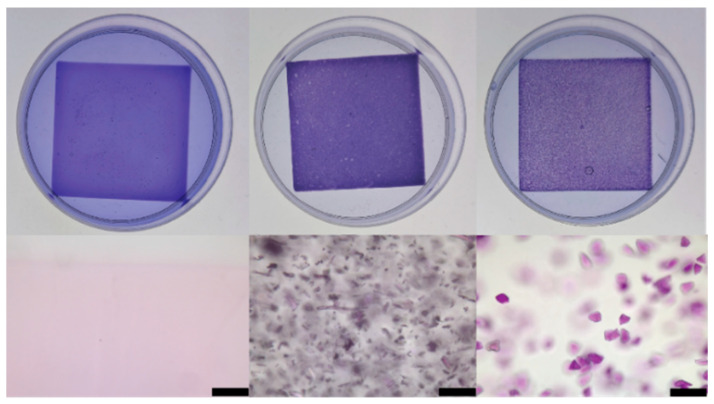
(**Top row**) photographs of three gels immersed in crystal violet solutions after the gel adsorption experiments. Length of gel samples is 4.8 cm. (**Bottom row**) transmitted light micrographs of a cross-section of each gel above. Black scalebars at bottom right are 200 µm. (**Left**) 2% by mass agarose; (**Center**) 2% by mass agarose + 1% by mass MC; (**Right**) 2% by mass agarose + 1% by mass SG.

To assess the adsorption capability of gelled agarose, gels were placed into a CV solution, and the color changes of the gel and solution were monitored over 90 min. Photographs of the gels after these experiments are displayed in Figure 3. In all cases, the gels became increasingly purple as the concentration of CV in solution decreased, an observation we attribute to both diffusion and adsorption. If adsorption played no role in dye migration through the gel, the gel and solution would equilibrate to the same CV concentration via diffusion. However, for all agarose gels studied, the gels became more purple than the solution in which they were immersed. All the gels tested, even those without added adsorbent, adsorb CV. The adsorption is attributed to the solid-like agarose network of the gel. Transmitted light micrographs of gel cross-sections, also displayed in Figure 3, show colocalization of the CV with the added adsorbent, showing that the additives also actively adsorb CV. They are removing CV from the solution that is diffusing throughout the gel matrix.

**Figure 4:**
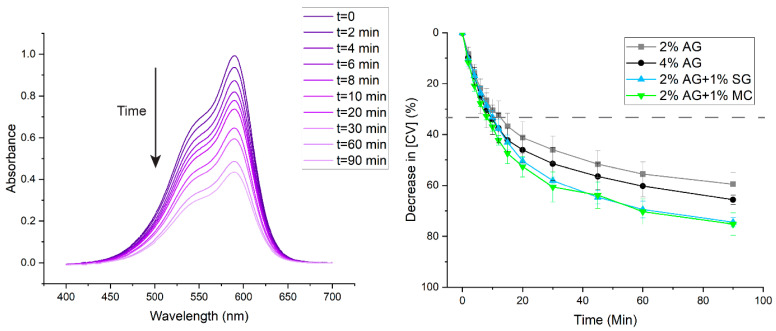
(**Left**) UV–Vis spectra of a crystal violet-containing bath as a function of time of agarose gel (AG) immersion. (**Right**) The percent decrease in crystal violet concentration in the bath as a function of time. Error bars show standard deviation of measurements on three different samples of the same formulation. The dashed gray line marks the maximum [CV] percent decrease if dilution from added water from the hydrogel were the only process involved. The [CV] percent decrease greater than (i.e., at higher values, below the line) this dashed line is attributed to adsorption.

The uptake of CV by the gel systems immersed in solution was monitored as a function of time with UV–Vis absorption spectroscopy. Absorption spectra, collected from a CV solution with an immersed 2% by mass agarose gel, are displayed in Figure 4 (left). The percent decrease in the concentration of CV, with 0% representing no change to the solution and 100% representing complete removal of CV from solution, is plotted as a function of time for a 2% by mass agarose gel, with and without additional adsorbent, and a 4% by mass agarose gel (Figure 4 (right)). No swelling of the agarose gel was measured upon placing the gel in water, so we can assume the CV concentration gradient is the main driving force for diffusion (as described by Fick’s law of diffusion [18]). From diffusion-led dilution alone, the CV distributes to an equilibrium concentration between the gel and the solution, so that the concentration of crystal violet [CV], determined from mass (m) and volume (v), in the bath would be expected to decrease from

(1) $${\left[CV\right]}_{initial}=\frac{m_{cv}}{v_{bath\;H_{2}O}},$$

to

(2) $${\left[CV\right]}_{final}=\frac{m_{cv}}{v_{gel\;H_{2}O}+v_{bath\;H_{2}O}},$$

so that the concentration decreases as

(3) $$\left(1-\frac{{\left[CV\right]}_{final}}{{\left[CV\right]}_{initial}}\right)\times 100=\%\;dilution.$$

In our experiments, the addition of approximately 5 mL of water in a gel ($v_{gel\;H_{2}O}$) to 10 mL of water in solution ($v_{bath\;H_{2}O})$ would result in a maximum concentration decrease of approximately 33% (represented by the dashed line in Figure 4) if dilution were the sole contributor to the concentration of CV in the solution. However, after 90 min, the 2% by mass agarose gel removed 59% of the CV from solution, and the 4% by mass agarose gel removed 66%. The 2% by mass agarose gels containing 1% by mass MC or SG adsorbent removed 75% CV in the same length of time. The amounts of CV removed as a function of time for these four gels are shown in Figure 4. The [CV] decrease in solution is attributed to a combination of dilution by water added from the gel (into which the CV diffuses) and adsorption in the gel, with all decreases in [CV] greater than 33% attributed to adsorption in the gel. Even after 90 min, the systems had not reached the maximum capacity for CV adsorption. Ultimately, diffusion throughout the gel is slow, and a typical paper cleaning conservation treatment is completed before this equilibrium state is reached. However, the tested gels remove a large amount of CV from the solution in the initial stages of these experiments (e.g., almost half the CV in the first 20 min), a time period of cleaning during which efficient extraction of stain materials is critical to mitigate treatment risks associated with prolonged exposure to water. Moreover, the addition of SG and MC to the 2% by mass agarose gel led to an increase in the uptake of CV from solution. The CV removal efficacy of these modified gel systems, compared to the agarose gel with no added adsorbent, exhibit a statistically significant increase (see Table S1).

### 2.2. Physical Property Measurements

**Figure 5:**
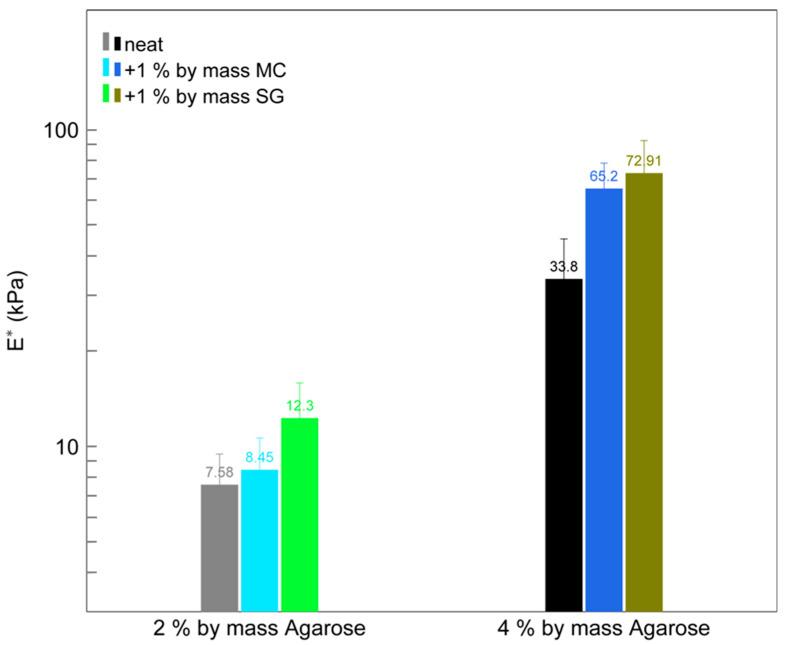
Effective modulus (E*) determined from static compression tests for agarose hydrogels with and without 1% by mass adsorbent added. Error bars show the standard deviation of measurements on four different spots on one sample.

Ideally, any modification to a gel should not induce changes that significantly alter the handling properties of the material in a detrimental way for the art conservator. Physical property changes induced by adding adsorbents were studied by measuring the elastic moduli of agarose gels upon incorporation of MC and SG. Specifically, the effective elastic modulus E* was measured for 2% by mass and 4% by mass agarose with and without added adsorbents. See the Supplementary File and Figure S1 for calculation details. Upon the addition of SG or MC to the gel, a slight increase in stiffness is observed (Figure 5). We speculate that the solid particles interact with the overall gel network and contribute to an increase in the strength of the network in relation to compressive forces. The reinforcement of hydrogels through the addition of a solid filler has been previously reported [19]. The addition of 1% by mass MC or SG leads to a small (~2×) increase in E*. The changes in handling properties, as far as conservation practice is impacted, are subtle. The gel with SG was noted by conservators to be slightly stiffer and more brittle. An increase in stiffness can decrease contact with a textured surface [20,21]. An increase in brittleness increases the risk of gel crumbs and/or particles of the added adsorbent remaining on the surface after cleaning. No remnants were noted in these experiments (Ideally no residue would be left behind by a conservation treatment, but the materials used here were chosen in part due to the fact that alterations are not expected if residual agarose, MC, or SG is left on paper after treatment. Silica gel is chemically inert under normal conditions, and the cellulose powder used is highly purified, 100% cellulose. However, we recommend inspection and removal of all potential gel and adsorbent remnants to avoid unintended consequences, as residue studies have not as of yet been performed.).

### 2.3. Water Loss Measurements

Generally, increasing the concentration of agarose in an agarose gel increases the water retention of the resulting gel [22]. This increase in water retention has been attributed to the decrease in pore size as the concentration of agarose is increased. For example, the average pore size, as measured with cryo-scanning electron microscopy, of agarose gels decreases from 230 nm to 150 nm as the concentration is increased from 1% to 2% [23]. An increase in the agar concentration from 3% to 5% produces a gel that is three times more water retentive, as measured in contact with absorbent stones [24].

**Figure 6:**
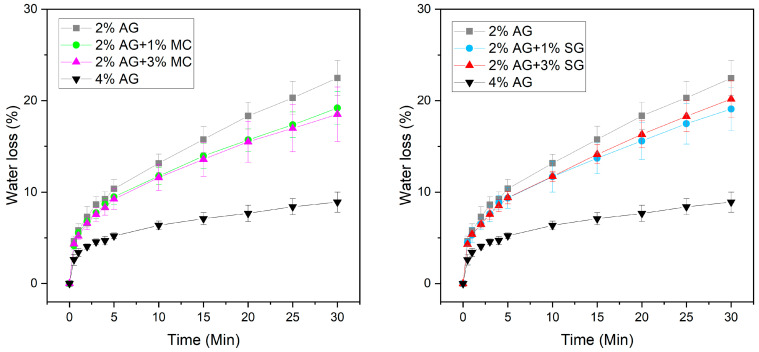
Percent water loss from an agarose (AG) gel, with either MC (**Left**) or SG (**Right**) added, as a function of contact time with Whatman No. 1 filter paper. Error bars designate standard deviation of measurements on three different samples of the same formulation.

To compare the water retention capability of agarose gels with and without adsorbent added, water delivery measurements were performed by measuring the mass loss into Whatman No. 1 filter paper as a function of contact time with the gel (Figure 6). Whatman No. 1 filter paper served as a highly absorbent testing material made from cotton linters with 98% alpha cellulose content, and is much more absorbent than art, writing, or printing papers [25,26]. A 2% by mass agarose gel (covered to minimize evaporation) delivered 23% by mass of its total water in 30 min, whereas the 4% by mass agarose gel only delivered 9% by mass of its total water in the same amount of time. The agarose gels that contain MC or SG at either 1% or 3% by mass lost approximately 20% by mass of total water into the paper. Although the addition of adsorbents provides a small but measurable increase in water retention, this increase does not appear to scale with the concentration added. Unfortunately, the addition of these adsorbents does not provide the ability to tailor water retention. However, these experiments do provide a route for material exploration: if we can identify additives that provide the ability to tune water retention, then gels could easily be tailored for use on specific papers of varying absorbencies on a case-by-case basis, without changing the concentration of gelator. This is helpful because agarose gels with high concentrations of agarose can be difficult to cast due to the high viscosity of the hot sol. Moreover, a gel formulation that requires less agarose is more economical and more sustainable. Agarose is a naturally derived polymer, and overharvesting has impacted global supply [27].

### 2.4. Comparative Gel Cleaning of 19th-Century Book Paper

Although these experiments have shown that adsorbent-containing gels are capable of increased adsorption of CV, the ability to also aid in the cleaning of paper containing water-soluble degradation products is key to their applicability in conservation. Many paper degradation products are colorless, such as glucose, acetic acid, oxalic acid, citric acid, and furfural; however, colored chromophore-containing compounds can be formed from oxidation, hydrolysis and Maillard reactions to include degraded carbohydrates, resins, and proteins, as well as other products containing conjugated bonds, such as certain lignin derivatives [26]. Localized introduction of water can induce the formation of tidelines, caused by the migration of components at the wet/dry interface that can induce degradation via localized oxidation of cellulose [28,29,30,31]. Washing yellowed and/or tideline-stained paper with water allows for the solubilization and removal of some of these colored compounds. A comparative study of washing artificially aged papers suggests that washing lightly discolored papers produces a larger color change than washing highly aged, browned papers [25]. Only a certain proportion of colored compounds in degraded paper are soluble in water, and this work seeks to provide a method to increase the removal efficacy of such compounds using an agarose gel.

**Figure 7:**
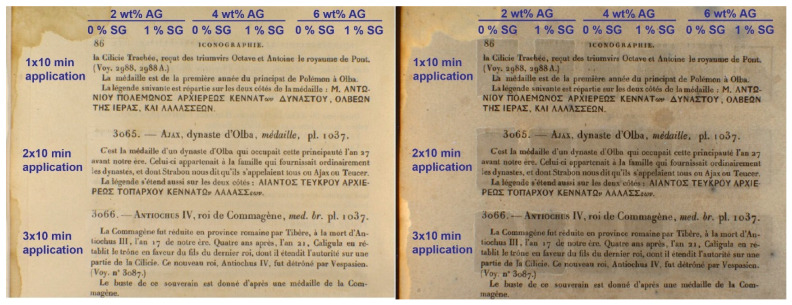
Visible light (**Left**) and ultraviolet-induced emission (**Right**) (365 nm λ_exc_; 400 nm–700 nm λ_em_) images of an 19th-century book page after localized gel treatment with 2%, 4%, and 6% by mass agarose (AG) gels with and without 1% by mass silica gel (SG) incorporated into the gel.

To assess the potential advantages of adsorbent-containing gels for cleaning stains on paper, localized gel tests were performed on a 19th-century book page. The page originates from a book published in 1853 by the Imprimerie Royale in Paris. The paper is machine-made laid, likely comprising rag fiber composition and sized with gelatin. The use of adsorbent-containing gels for localized treatment of paper was examined via a series of tests with agarose gels of 2%, 4%, and 6% by mass agarose with and without 1% by mass SG placed for 10 min applications either one, two, or three times in the same location (Figure 7). The cleaned areas appear brighter when viewed with visible light, especially for 2% and 4% by mass agarose gels. However, the effects are subtle, and any differences are difficult to note with visible light alone. Because some of the paper degradation products (such as aged gelatin sizing) fluoresce under UV light, viewing under UV light allows for better comparisons between cleaned areas [10]. The areas cleaned with 1% by mass SG incorporated into 2% or 4% by mass agarose appear slightly darker than those areas cleaned with gels without SG, indicative of more effective cleaning.

The effects of varying water retention among the different gel formulations are evident in the appearance of the treated areas after cleaning. All the areas cleaned using 6% by mass agarose gels (with and without SG) were cleaned unevenly. Visual observations made during the course of the experiment suggest that the gel did not release enough water to wet the paper evenly and the surface contact was poor. On the other hand, all the 2% by mass agarose gels (with and without SG) released too much water, leading to the formation of tidelines induced by the migration of solubilized material through the paper that was not picked up by the gel. The 4% by mass agarose gels wet the surface evenly enough for homogenous cleaning across the surface of the paper but did not release enough water to induce visible tidelines. These 4% by mass agarose gel formulations are well suited to this particular paper’s moderate absorbency. The results from the cleaning of a more absorbent paper, in this case a book page from the late-19th century, are presented in Figure S2, showing that a 6% by mass agarose gel was required to prevent visible tidelines.

Although the water retention measurements on Whatman filter paper displayed in Figure 6 showed a slight increase in water retention upon the incorporation of 1% by mass SG into the agarose gels, this small increase does not appear to lead to an appreciable reduction in the development of tidelines in Figure 7 after cleaning with the 2% by mass agarose gel formulations. Further modifications to the gel formulation that allow better control over water delivery would be an improvement to the gel cleaning applications used in conservation.

### 2.5. Gel Cleaning of a Water-Damaged 18th-Century Print with an MC-Containing Agarose Gel

**Figure 8:**
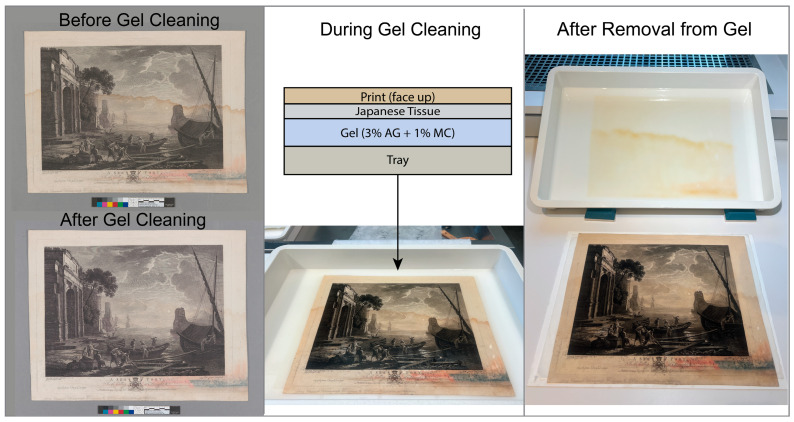
*A Sea Port*, After Claude Lorrain, 1775. National Gallery of Art non-accessioned study collection. Images show different stages of the overall microcellulose-containing agarose gel cleaning of the 18th-century print *A Sea Port*. The still-wet print is shown in the lower right of the image, and the print after drying is shown in the lower left.

To gather insight into how the incorporation of adsorbents into agarose gels could be a method implemented in conservation practice, a case study was performed on *A Sea Port*, an 18th-century intaglio print on lightly sized and therefore absorbent wove paper. The print selected contained a tideline spanning the entire length of the paper (Figure 8). Tidelines left untreated on artworks induce uneven aging and complex degradation pathways within the paper [30,32]. Although nothing is known about the event that induced this tideline, its presence indicates that water-soluble components—consisting of those already present in the paper as well as those present in the water introduced to the print—migrated throughout the paper. Any components that remain water-soluble should be readily solubilized and extracted with a water-based gel. To begin the treatment, the surface of the print was cleaned using a cosmetic sponge. Gel cleaning was carried out using a 3% by mass agarose and 1% by mass MC hydrogel. After casting the gel into a tray and cooling, a water-permeable Japanese tissue barrier was laid directly on the gel, serving primarily as a support for handling the print when wet, and finally, the humidified print was placed in the tray. A schematic of the cleaning process is displayed in Figure 8 alongside photographs collected before, during, and after cleaning. The print was cleaned with the gel and tissue in contact with the back of the print so that the image material could be monitored through the cleaning. It should be noted, however, that as the water stain penetrates the entire paper thickness, cleaning from either or both sides may be appropriate as long as the image material is stable. More photographs are presented in Figure S3. As evidenced by visual inspection of the gel after treatment, a one-hour gel cleaning of the print induced the solubilization and uptake of yellowed degradation products from the entire sheet, including the tideline and to both sides of it. The print after gel cleaning and drying was lighter overall, and the tideline was significantly reduced. The appearance of the print was substantially improved without any use of bleaching agents or chelators.

Incorporating MC is a simple modification to the formulation. Once cast, there is no change in the conservation methodology compared to a gel without MC. Although the optical clarity of the gel decreases with the addition of MC, a clear gel is not critical to this method for overall treatment, as gel cleaning is typically performed from below rather than above.

**Figure 9:**
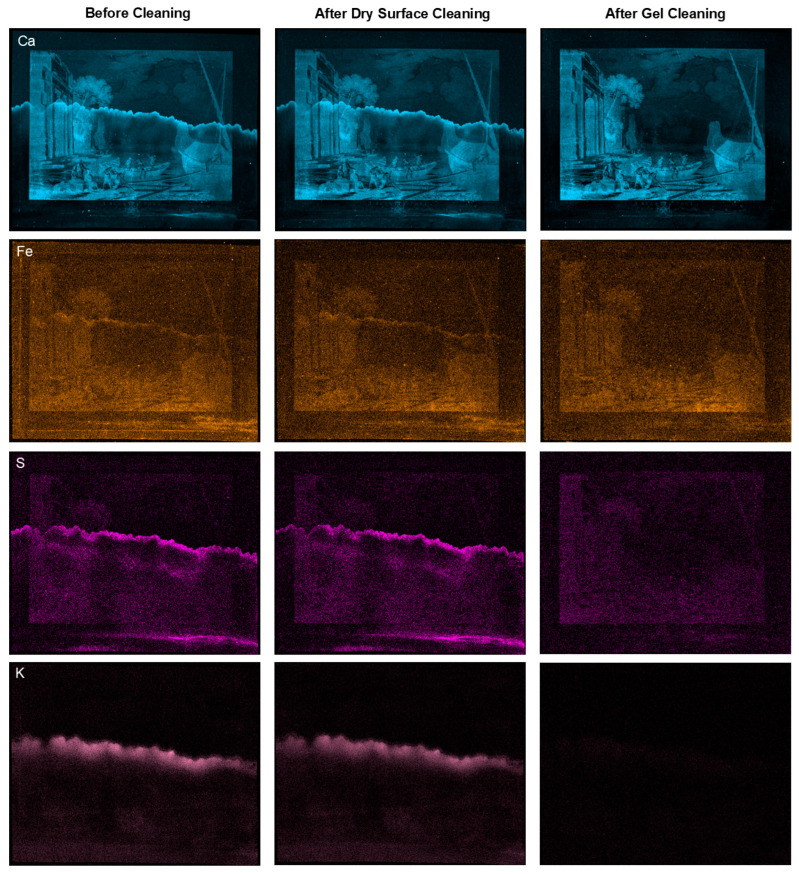
Element maps show the distribution of calcium (Ca), iron (Fe), sulfur (S), and potassium (K) in the print before cleaning, after dry surface cleaning, and after gel cleaning with a 3% by mass agarose hydrogel with 1% by mass MC. Maps for a single element are presented on the same intensity scale for each stage of the treatment. Among the different elements, the map color intensities represent relative scaling and concentration.

**Figure 10:**
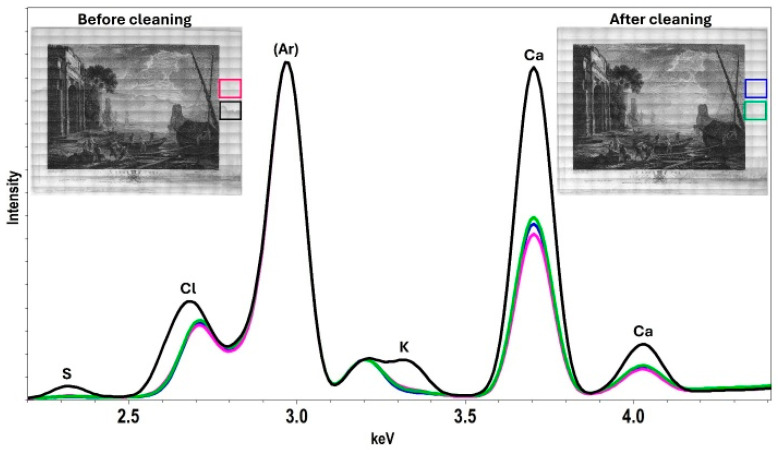
Reconstructed XRF spectra showing the presence of elements above and at the tideline before and after gel cleaning. Before gel cleaning, the tideline (black) contains calcium (Ca), sulfur (S), chlorine (Cl), and potassium (K). A spectrum of the tideline collected after gel cleaning (green) with a 3% by mass agarose hydrogel with 1% by mass MC shows a reduction in the amounts of these elements. Additionally, comparison of spectra collected from an area above the tideline (pink and blue) show negligible change in the element composition before and after treatment.

X-ray fluorescence (XRF) mapping was used to characterize the distribution of elements throughout the print and its paper support at three treatment stages: before treatment, after dry surface cleaning, and after gel cleaning (Figure 9). The resulting element maps obtained from fitting the XRF data visualize the spatial distribution of detected elements, and comparison of the before and after-treatment maps shows some of the changes resulting from the treatment. The tideline is clearly discernable in the before-treatment maps for calcium (Ca), iron (Fe), sulfur (S), and potassium (K). Large signals from calcium, potassium, and sulfur in the tideline suggest that large proportions of these elements were introduced to the paper when the tideline was formed. Maps collected after dry cleaning show that this cleaning method reduced surface iron, especially near the edge of the paper, but had no discernable effect on the tideline. Element maps and reconstructed XRF spectra (Figure 10) collected after gel treatment show a large reduction or complete removal of the elements associated with salts present in the tideline. These results demonstrate that effective removal of the salts required a water-based cleaning method. The use of an adsorbent-containing gel provided a simple and effective method for improving the appearance and removing degradative components from the print.

XRF maps of calcium and iron show that the presence of these elements in the paper is not discernably changed by the treatment. This observation is confirmed by reconstructed XRF spectra collected from an area of paper above the tideline (Figure 10), which show very little change in the amount of calcium before and after gel cleaning. Spectra collected directly on the tideline reveal that gel cleaning reduced the amounts of elements associated with the tideline (Ca, Cl, K, S) without considerably changing the amounts of elements in the paper. Similarly, element maps of the ink before treatment reveal the presence of calcium, phosphorus, and iron. After gel treatment maps show no apparent change in the elemental composition of the ink, suggesting it was unaffected by the gel cleaning.

The distributions of some elements are more diffuse than others, evidence for differential migration of soluble salts through the paper. For example, the tideline visualized in the before-treatment calcium map is much thinner (less diffuse) than the tideline in the sulfur and potassium maps. Additionally, before-treatment maps show potassium, chlorine, and bromine located along the entire bottom edge of the print. This element distribution is consistent with compounds that may have been deposited when the print was submerged in contaminating water but did not migrate up the paper along with the salts present in the tideline. These compounds were successfully reduced or removed by gel treatment. Overall, the maps show that many of the elements are present in multiple species with different polarities and solubilities, yet gel cleaning effectively removed all types from the paper support.

## 3. Conclusions

A hydrogel placed on paper loses some of its water to the paper, ideally allowing for the solubilization of discoloration. If too much water is delivered by a locally placed gel, solubilized material travels laterally within the paper, inducing tidelines. However, if just enough water is delivered to lightly but evenly wet the paper and solubilize the stain material, then much of the solubilized material can diffuse into the gel. At this point in the gel cleaning process, adsorption of stain components to the gel network can take place. Here, we show that adsorption is a key factor in gel cleaning. As solubilized material is absorbed into a gel during cleaning, the process of adsorption enables the sequestration of solubilized material, thereby preventing the redeposition of solubilized material and increasing cleaning efficacy.

A more thorough understanding of properties involved in gel cleaning allows conservators to tailor gels for more effective cleaning. Here, incorporating additional adsorbent into the gel provided a simple, affordable method to increase the amount of material onto which solubilized material can adsorb, thereby increasing cleaning efficacy. Agarose can adsorb solubilized material from degraded paper, and many other gel systems may exhibit this type of behavior as well. For systems in which the gel network itself does not efficiently adsorb certain ions or molecules, the addition of adsorbents would serve to significantly increase efficacy. Because adsorbents can be easily incorporated into gels, they can be chosen selectively as is best suited to the challenges of specific cleaning goals. Specifically, for cleaning stains from degraded materials, the choice of adsorbent is influenced by the nature of the degradation products. Overall, this research suggests a strategy for conservation treatment material innovation: by exploring the physical processes that are involved in methodologies that are successfully and routinely used by art conservators, we can devise new formulations to improve the tools available to art conservators.

## 4. Materials and Methods

### 4.1. Materials

Agarose (Sigma-Aldrich, St. Louis, MO, USA, Type I, low EEO), microcrystalline cellulose (Solka Floc 300 FCC powdered cellulose, North Tonawanda, NY, USA), silica gel (Aldrich, St. Louis, MO, USA, 200–400 mesh, 60 Å), crystal violet (Eastman, Kingsport, TN, USA, 99%), and Amberlite XAD7HP (Sigma-Aldrich, 20–60 mesh) were used as received. Water (18 MΩ), unless otherwise noted, was filtered through an Elga PURELAB^®^ Quest water purification system (High Wycombe, UK).

### 4.2. Methods

#### 4.2.1. Gel Preparation

Gels consisting of agarose concentrations ranging from 1% to 10% are employed in conservation, depending on the application. Here, formulations comprising 2%, 4%, and 6% agarose were selected to systematically assess how variations in porosity, and the related process of water release, affects cleaning with and without adsorbents. Gels with larger proportions of agarose consist of a smaller mean pore size thereby release less water upon contact with an absorbent surface like paper. Agarose (2%, 4%, or 6% by mass), adsorbent (microcellulose or silica gel, 0%, 1%, or 3% by mass), and water were combined in a glass vial sealed with a screw-top lid. The mixture was heated in an 85 °C water bath until dissolved (approximately 5 min). The hot solution was poured into a silicone mold with dimensions of 4.8 cm × 4.8 cm and gently swirled until an increase in viscosity was visually noted (approximately 1 min). This step prevented adsorbent from settling. Gel casting was performed at room temperature (21 °C) and 50% relative humidity. The gels were covered to slow evaporation and were allowed to rest at room temperature for at least 1 h before experiments.

#### 4.2.2. Solid Adsorption Kinetics Measurements

50 mg of an adsorbent was placed into a vial with a screw-top cap. 2.7 g of aqueous 3 × 10^−5^ M crystal violet (pH measured to be 5.5) was added to the vial. The vial was capped and shaken by hand for 5 s. After 1 h, an aliquot of the solution was placed in a poly(methyl methacrylate) cuvette, and a UV–Vis absorption spectrum was collected from 400 nm to 700 nm with a Cary 1G UV-Visible Spectrophotometer. These experiments were performed under laboratory conditions (21 °C, 50% RH).

#### 4.2.3. Gel Adsorption Kinetics Measurements

Each gel sample was weighed and subsequently added to 10 mL of aqueous crystal violet solution (3.0 × 10^−5^ M) in a plastic Petri dish. UV–Vis spectroscopy was used at specific periods of time to measure the absorbance of aliquots of the solution, which were then returned to the Petri dish. Petri dish, with cover placed to slow evaporation, was swirled every few minutes to minimize concentration gradients in the crystal violet solution. These experiments were performed under laboratory conditions (21 °C, 50% RH). ANOVA statistical analysis was performed, and Tukey test results are presented in the Supporting Information Table S1.

#### 4.2.4. Elastic Modulus Measurements

Static compression tests were performed to determine the effective elastic modulus (E*), a measurement of stiffness, of the gels with and without added adsorbents [33,34]. A glass half-ball lens (B270, Edmund Optics, Barrington, NJ, USA) of either 3 mm (17 mg), 4 mm (42 mg), 5 mm (80 mg), or 6 mm (149 mg) in diameter was weighed and placed on the surface of a gel. These experiments were performed under laboratory conditions (21 °C, 50% RH). Microscope images of the contact area (=*pa^2^*) between the lens and the gel were obtained with a Leica DM6 M microscope. By measuring the contact radius (*a*) and using the half-ball lens’ radius (*R*), the effective elastic modulus (E*) was determined with Equation (4) [35,36,37,38,39]. Details of the derivation of Equation (4) and the extrapolation procedure for the effective modulus are provided in the Supporting Information.

(4) $$F_{N}\approx -F_{adh}+\frac{4}{3}\frac{E^{*}a^{3}}{R}$$

#### 4.2.5. Water Retention Measurements

A gel weighing approximately 5 g was placed for 1 s on a lint-free wipe to remove any potential water droplets on the surface of the gel. The gel was weighed and then placed on Whatman filter paper (No. 1) for 30 s, after which it was weighed again. This experiment was repeated at time intervals across 30 min of total contact time. The top surface of the gel was covered with an inverted Petri dish cover to slow evaporation and improve contact between the gel and paper. The percent water retention was determined from the weight of the gel at each measurement in relation to the total weight of water in the gel. These experiments were performed under laboratory conditions (21 °C, 50% RH).

#### 4.2.6. Localized Gel Treatment of Book Page

Cast and cooled gels were cut into 2.25 cm × 1.5 cm slabs and placed on a 19th-century book page for 10 min intervals with either one, two, or three applications in the same location, allowing the page to fully dry before subsequent applications. The book page was allowed to dry and imaged with visible and ultraviolet illumination with a Foster + Freeman VSC 8000/HS workstation. The camera filter for the 365 nm ultraviolet illumination filters out wavelengths below 400 nm.

#### 4.2.7. Overall Gel Treatment of Print

Initial solubility testing of inks and graphite inscriptions using deionized water revealed no water sensitivities. Surface cleaning was performed using non-latex cosmetic sponges on both recto and verso, followed by localized reduction in heavy soiling with Mars Vinyl eraser blocks, with the image area excluded from surface cleaning. The print was humidified in a closed chamber until the paper became limp, then misted with deionized water to fully relax the sheet. Following the gel formulation as described above, the print was positioned over the pre-cast gel, with pre-washed Sekishu Japanese paper as an intermediary barrier layer to facilitate safe removal post-treatment. Japanese paper was selected over polyester webbing as it permits unrestricted movement of water and degradation products while providing superior contact and minimizing air bubble formation between the gel and object. After placement, the print was misted from above with deionized water and even contact was achieved using a Noribake brush through lightweight polyester webbing Hollytex (Ahlstrom, Espoo, Finland). The webbing was then removed, and the print remained in contact with the gel for one hour, with periodic misting to prevent uneven migration of stain materials and premature drying. The print was then lifted and passively air-dried for approximately one hour before being dried and flattened under moderate pressure between wool felts for two weeks. The deionized water used for the treatment was purified from tap water using a Barnstead B-Pure 3-Module Water Purification System (Thermo Scientific, Waltham, MA, USA) equipped with a digital resistivity meter reading between 15 MΩ-cm–16.5 MΩ-cm. Although calcium-modified water would be used during a typical conservation treatment to replace calcium removed with washing, the decision to use water without added calcium was made so that the calcium distribution within the paper could be monitored with XRF as a function of cleaning.

#### 4.2.8. X-Ray Fluorescence Mapping

Qualitative XRF mapping was used to characterize the print before and after treatment to show the element distribution of the artist’s materials and the tideline. A Bruker M6 Jetstream (Billerica, MA, USA) was equipped with a rhodium (Rh) anode X-ray tube, two 60 mm^2^ silicon drift detectors, and a helium purge. The X-ray tube voltage was 35 kV, the current was 800 μA, the spot size was 580 μm, the pixel size was 580 μm, and the acquisition time was 40 ms/pixel.

## Figures and Tables

**Table 1:** Percent removal of crystal violet from a 3 × 10^−5^ M solution upon mixing with 50 mg of various compounds, as measured with UV–Vis absorption spectroscopy after 1 h. Error bars designate standard deviation of measurements collected from three different samples.

Material Added to CV Solution	CV Removal in 1 h (%)
Silica gel powder (SG)	94 ± 1
Microcellulose powder (MC)	93 ± 5
Agarose	90 ± 3
Whatman filter paper	59 ± 3
Amberlite XAD7HP	45 ± 8
Silica gel desiccant	22 ± 3
Control (glass vial; no adsorbent)	14 ± 5

## Data Availability

Dataset available on request from the authors.

## Supplementary Materials

The following supporting information can be downloaded at: https://www.mdpi.com/article/10.3390/gels11120965/s1, Figure S1: Estimating the elastic modulus of the agarose gels as a function of gel formulation; Figure S2: Visible (left) and 368 nm ultraviolet-induced (right) light images of a late-19th century book page after localized gel treatment with 2%, 4%, and 6% by mass agarose gels with 1% by mass microcellulose (MC) incorporated into the gel network; Figure S3: *A Sea Port*, After Claude Lorrain, 1775. National Gallery of Art non-accessioned study collection. Images showing different stages of the overall dry surface cleaning and microcellulose-bulked agarose gel cleaning of the 18th-century print *A Sea Port.* Table S1: Results of a Tukey test used for statistical analysis. Sig equals 1 indicates that the difference in the means is significant at the 0.05 level. Sig equals 0 indicates that the difference in the means is not significant at the 0.05 level.

## Abbreviations

The following abbreviations are used in this manuscript:

AG	Agarose
Br	Bromine
Ca	Calcium
Cl	Chlorine
CV	Crystal violet
Fe	Iron
K	Potassium
MC	Microcellulose
S	Sulfur
SG	Silica gel
UV–Vis	Ultraviolet/visible
XRF	X-ray fluorescence

## References

[1] Dwan A. (1987). Paper Complexity and the Interpretation of Conservation Research. J. Am. Inst. Conserv..

[2] Whitmore P.M., Banik G., Bruckle I. (2011). Paper Aging and the Influence of Water. Paper and Water: A Guide for Conservators.

[3] Zervos S., Alexopoulou I. (2015). Paper conservation methods: A literature review. Cellulose.

[4] Van Der Reyden D. (1992). Recent Scientific Research in Paper Conservation. J. Am. Inst. Conserv..

[5] Warda J., Brückle I., Bezúr A., Kushel D. (2007). Analysis of Agarose, Carbopol, and Laponite Gel Poultices in Paper Conservation. J. Am. Inst. Conserv..

[6] Khaksar-Baghan N., Koochakzaei A., Hamzavi Y. (2024). An overview of gel-based cleaning approaches for art conservation. Herit. Sci..

[7] Domingues J.A., Bonelli N., Giorgi R., Fratini E., Gorel F., Baglioni P. (2013). Innovative hydrogels based on semi-interpenetrating p(HEMA)/PVP networks for the cleaning of water-sensitive cultural heritage artifacts. Langmuir.

[8] Mazzuca C., Micheli L., Carbone M., Basoli F., Cervelli E., Iannuccelli S., Sotgiu S., Palleschi A. (2014). Gellan hydrogel as a powerful tool in paper cleaning process: A detailed study. J. Colloid Interface Sci..

[9] Sullivan M.R., Duncan T.T., Berrie B.H., Weiss R.G., Angelova L.V., Ormsby B., Townsend J.H., Wolbers R. (2017). Rigid Polysaccharide Gels for Paper Conservation: A Residue Study. Gels in the Conservation of Art.

[10] Henniges U., Brückle I., Khaliliyan H., Böhmdorfer S. (2024). Gellan residues on paper: Quantification and implication for paper conservation. Herit. Sci..

[11] Franco S., Severini L., Buratti E., Tavagnacco L., Sennato S., Micheli L., Missori M., Ruzicka B., Mazzuca C., Zaccarelli E. (2025). Gellan-based hydrogels and microgels: A rheological perspective. Carbohydr. Polym..

[12] Severini L., Tavagnacco L., Angelini R., Franco S., Bertoldo M., Calosi M., Micheli L., Sennato S., Chiessi E., Ruzicka B. (2023). Methacrylated gellan gum hydrogel: A smart tool to face complex problems in the cleaning of paper materials. Cellulose.

[13] Di Napoli B., Franco S., Severini L., Tumiati M., Buratti E., Titubante M., Nigro V., Gnan N., Micheli L., Ruzicka B. (2020). Gellan Gum Microgels as Effective Agents for a Rapid Cleaning of Paper. ACS Appl. Polym. Mater..

[14] Hughes A., Sullivan M. (2016). Targeted Cleaning of Works on Paper: Rigid Polysaccharide Gels and Conductivity in Aqueous Solutions. Book Pap. Group Annu..

[15] Seow W.Y., Hauser C.A.E. (2016). Freeze–dried agarose gels: A cheap, simple and recyclable adsorbent for the purification of methylene blue from industrial wastewater. J. Environ. Chem. Eng..

[16] Guastaferro M., Baldino L., Cardea S., Reverchon E. (2022). Different Drying Techniques Can Affect the Adsorption Properties of Agarose-Based Gels for Crystal Violet Removal. Appl. Sci..

[17] Maqbool Q., Cavallini I., Lasemi N., Sabbatini S., Tittarelli F., Rupprechter G. (2024). Waste-Valorized Nanowebs for Crystal Violet Removal from Water. Small Sci..

[18] Perullini M., Jobbagy M., Japas M.L., Bilmes S.A. (2014). New method for the simultaneous determination of diffusion and adsorption of dyes in silica hydrogels. J. Colloid Interface Sci..

[19] Dellatolas I., Bantawa M., Damerau B., Guo M., Divoux T., Del Gado E., Bischofberger I. (2023). Local Mechanism Governs Global Reinforcement of Nanofiller-Hydrogel Composites. ACS Nano.

[20] Duncan T.T., Chan E.P., Beers K.L. (2019). Maximizing Contact of Supersoft Bottlebrush Networks with Rough Surfaces To Promote Particulate Removal. ACS Appl. Mater. Interfaces.

[21] Duncan T.T., Chan E.P., Beers K.L. (2021). Quantifying the ‘press and peel’ removal of particulates using elastomers and gels. J. Cult. Herit..

[22] Bertasa M., Poli T., Riedo C., Di Tullio V., Capitani D., Proietti N., Canevali C., Sansonetti A., Scalarone D. (2018). A study of non-bounded/bounded water and water mobility in different agar gels. Microchem. J..

[23] Jayawardena I., Turunen P., Garms B.C., Rowan A., Corrie S., Grøndahl L. (2023). Evaluation of techniques used for visualisation of hydrogel morphology and determination of pore size distributions. Mater. Adv..

[24] Sansonetti A., Bertasa M., Canevali C., Rabbolini A., Anzani M., Scalarone D. (2020). A review in using agar gels for cleaning art surfaces. J. Cult. Herit..

[25] Vitale T., Erhardt D. Changes in paper color due to artificial aging and the effects of washing on color removal. Proceedings of the ICOM Committee for Conservation, 10th Triennial Meeting.

[26] Kosek J., I’Anson S., Tindal A., Daniels V., Sandy M. (2014). Model discoloured paper for analysis of aqueous washing and other conservation processes/Untersuchung und Bewertung wässriger Behandlungstechniken mittels gefärbter Modellpapiere/Analyse et évaluation de techniques de traitement aqueux grâce aux papiers modèles colorés. Restaur. Int. J. Preserv. Libr. Arch. Mater..

[27] Santos R., Melo R.A. (2018). Global shortage of technical agars: Back to basics (resource management). J. Appl. Phycol..

[28] Eusman E. (1995). Tideline Formation in Paper Objects: Cellulose Degradation at the Wet-Dry Boundary. Stud. Hist. Art.

[29] Dupont A.L. (1996). Degradation of Cellulose at the Wet/Dry Interface. I. The Effect of Some Conservation Treatments on Brown Lines. Restaur. Int. J. Preserv. Libr. Arch. Mater..

[30] Jeong M.-J., Dupont A.-L., de la Rie E.R. (2012). Degradation of cellulose at the wet-dry interface: I—Study of the depolymerization. Cellulose.

[31] Jeong M.J., Dupont A.L., de la Rie E.R. (2014). Degradation of cellulose at the wet-dry interface. II. Study of oxidation reactions and effect of antioxidants. Carbohydr. Polym..

[32] Souguir Z., Dupont A.-L., de la Rie E.R. (2008). Formation of Brown Lines in Paper: Characterization of Cellulose Degradation at the Wet−Dry Interface. Biomacromolecules.

[33] Shull K.R. (2002). Contact mechanics and the adhesion of soft solids. Mater. Sci. Eng. R Rep..

[34] Duncan T.T., Vicenzi E.P., Lam T., Brogdon-Grantham S.A. (2023). A Comparison of Materials for Dry Surface Cleaning Soot-Coated Papers of Varying Roughness: Assessing Efficacy, Physical Surface Changes, and Residue. J. Am. Inst. Conserv..

[35] Hertz H. (1881). Über die berührung fester elastischer Körper. J. Für Die Reine Angew. Math..

[36] Hertz H. (1882). Über die Berührung fester elastischer Körper und Über die Härte. Verhandlungen des Vereins zur Beförderung des Gewerbefleisscs.

[37] Johnson K.L. (1987). Contact Mechanics.

[38] Johnson K.L., Kendall K., Roberts A.D. (1971). Surface energy and the contact of elastic solids. Proc. R. Soc. Lond. A Math. Phys. Sci..

[39] Popov V.L., Heß M., Willert E. (2019). Handbook of Contact Mechanics.

