# Supplementary material for: The Role of Adsorption in Agarose Gel Cleaning of Artworks on Paper
Source: Gels. 2025 Nov 29;11(12):965. doi: 10.3390/gels11120965 (PMC12733167; doi:10.3390/gels11120965)
Supplement: Supplementary file 1 [file gels-11-00965-s001.zip › gels-3923737-supplementary.pdf]

The Role of Adsorption in Agarose Gel Cleaning of Paper Artworks

Teresa T. Duncan[1], Michelle R. Sullivan[2], Amy Hughes[3], Kathryn Morales[1], Edwin P. Chan[4], Barbara H. Berrie[5]

[1]Scientific Research, National Gallery of Art, 2000 South Club Drive, Landover, MD 20785, USA
[2]Department of Paper Conservation, J. Paul Getty Museum, 1200 Getty Center Drive, Los Angeles, CA 90049, USA
[3]Paper Conservation, National Gallery of Art, 2000 South Club Drive, Landover, MD 20785, USA
[4]Materials Science and Engineering Division, National Institute of Standards and Technology, Gaithersburg, MD 20899, USA

[5]Independent Resesarcher, Washington, DC 20010, USA

Email: t-duncan@nga.gov

1 Determination of the Elastic Modulus of a Gel

In the absence of adhesion and assuming that the elastic modulus of the lens is significantly greater than the gel ($E^*_\mathrm{p} \gg E^*$), the contact radius of the interface (a) is related to the radius of the lens (R) according to the Hertz model,[1] which relates the contact force due imposed by the lens (F_N) to the resistance to deformation by the gel

$$F_\mathrm{N} = \frac{4}{3}\frac{E^* a^3}{R} \ , \tag{1}$$

where $E^* = E/(1-\nu^2)$ is the plane-strain elastic modulus of the gel and ν is the Poisson's ratio. Note that this model assumes the mechanical properties of the gel are isotropic, which is typically assumed for most gels.[2] Because most gels are considered incompressible, we report E^* as opposed to E.

For this study, the contact force can be expressed as the force due to gravity of the lens ($F_\mathrm{N} = m_\mathrm{p} g$). Equation (1) can be further simplified by relating the mass of the half-ball lens in terms of its density (ρ_p) and R:

$$\begin{aligned} m_\mathrm{p} g &= \frac{4}{3}\frac{E^* a^3}{R} \\ \frac{2}{3}\pi\rho_\mathrm{p} g R^3 &= \frac{4}{3}\frac{E^* a^3}{R} \\ \implies R^4 &= \left(\frac{2E^*}{\pi\rho_\mathrm{p} g}\right) a^3 \ . \end{aligned} \tag{2}$$

Thus, the final expression relates the radius of the half-ball lens to the elastic modulus of the gel, the density of the lens, the gravitational constant, and the contact radius of the interface.

The density of the half-ball lens can be estimated from literature or can be directly measured. To directly measure the density, recognize that it is related to the radius of the half-ball lens as

$$F_\mathrm{N} = \left(\frac{2}{3}\pi\rho_\mathrm{p} g\right) R^3 \ , \tag{3}$$

where F_N is the weight of the lens and $g = 9.81$ m s^{-2} is the acceleration due to gravity. Specifically, by weighing the lens and measuring its radius, the density can be determined. Note that the volume of the half-ball lens is the volume of a hemisphere.

In the presence of adhesion, the additional adhesive force (F_adh) causes the contact radius to increase. The Hertz model can be modified to account for this additional force, which is the theory of Johnson, Kendall, and Roberts (JKR).[3] For simplicity, we assume that the force due to adhesion is not significant and does not scale with a. Under these assumptions, Equation (2) becomes

$$F_\mathrm{N} \approx -F_\mathrm{adh} + \frac{4}{3}\frac{E^* a^3}{R} \ . \tag{4}$$

From **Figure S1a**, we determined that $\rho_\mathrm{p} = 2577$ kg m^{-3}.

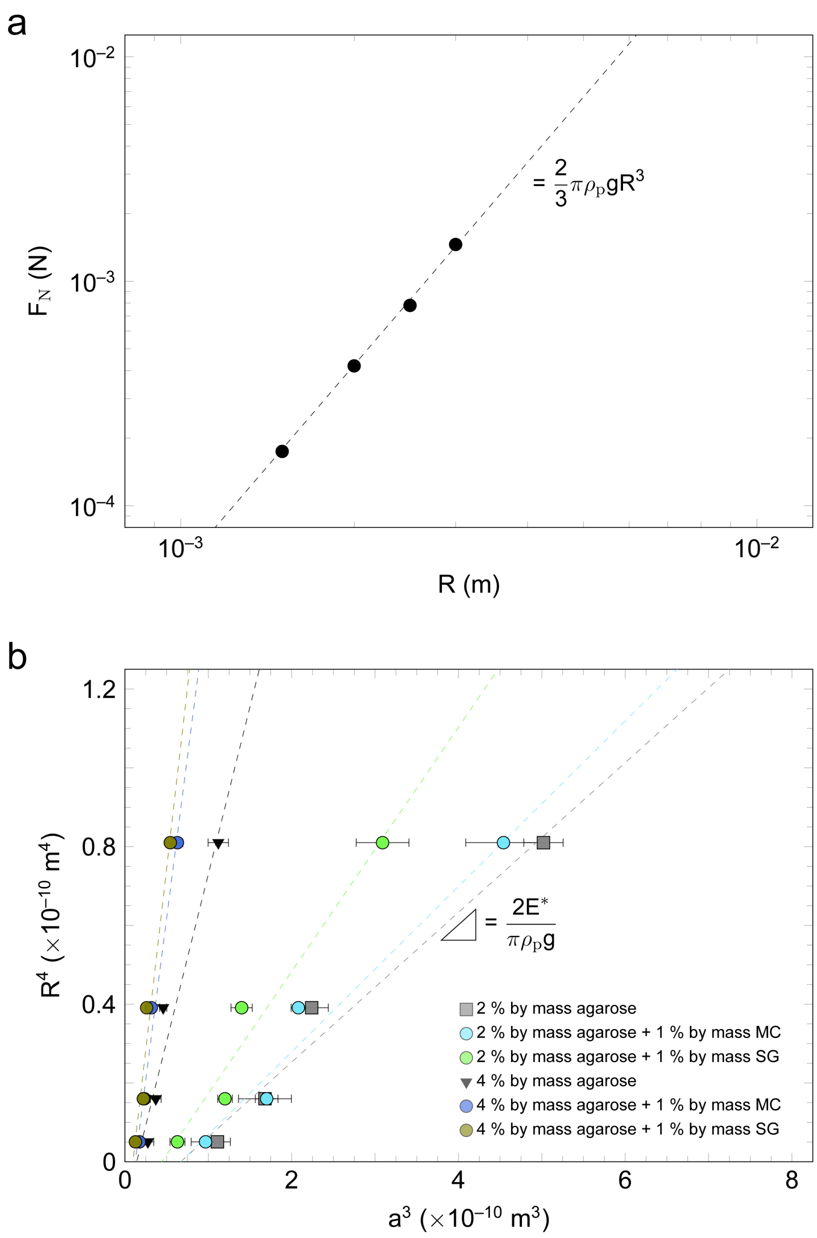

Figure S1: Estimating the elastic modulus of the agarose gels as a function of gel formulation. **a)** The density of the half-ball lens (ρ_p) is determined by plotting the contact force imposed by the lens on the gel (F_N) versus the radius of the half-ball lens (R). The slope of this plot is related to ρ_p. **b)** The plane-strain elastic modulus of the gel (E^*) can be determined by plotting R^4 versus a^3. The slope of this relationship is related to E^*.

Now that the density is determined, we can use Equation (4) to calculate the plane strain elastic modulus of the gel (E^*) by relating R to a. For simplicity, we can plot the data in terms of R^4 vs. a^3 and fit the data using the

linearized form of Equation (4),

$$
\begin{aligned}
F_{\mathrm{N}} &\approx -F_{\mathrm{adh}} + \frac{4}{3}\frac{E^{*}a^{3}}{R} \\
\implies R^{4} &\approx C + \left(\frac{2E^{*}}{\pi\rho_{\mathrm{p}}g}\right)a^{3}\ .
\end{aligned} \tag{5}
$$

To determine the elastic modulus (E^{*}) of a particular gel, we plot the experimental results as R^4 versus a^3. By applying Equation (5) to the results, the slope of the curve is $= 2E^{*}/(\pi\rho_{\mathrm{p}}g)$ and the $y-$intercept is C, which is a variable related to adhesion. Since ρ_{p} was previously measured and g is known, we can determine E^{*} from this method. As shown in **Figure S1b**, the experimental results are in good agreement with Equation (5).

We can make several comments about these results. First, the full solution for Equation (5) is the JKR theory,[3] which is a nonlinear function. Our linearized form simplifies the extrapolation procedure, but could potentially lead to accuracy issues if the adhesion energy relative to the elastic modulus is significantly large. This is evident when comparing the 2 % by mass agarose gel versus the 4 % by mass agarose gel where we find that better fits were observed for the 4 % by mass gels. Second, a pragmatic approach to circumvent using the full JKR model when adhesion is significant, i.e., when the gels are tacky, is to use large probes that increase F_{N}. This effectively reduces the effect of adhesion but also improves measurement accuracy of the contact radius.

2 Additional Figures

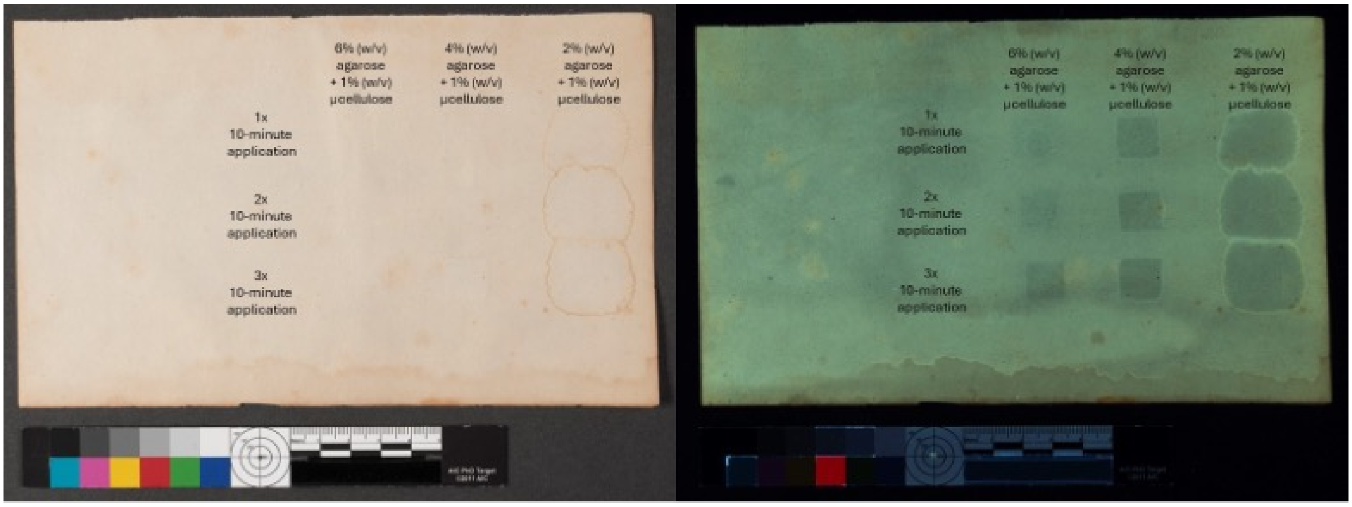

Figure S 2: Visible (left) and 368 nm ultraviolet-induced (right) light images of a late-19th century book page after localized gel treatment with 2 %, 4 %, and 6 % by mass agarose gels with 1 % by mass microcellulose (MC) incorporated into the gel network.

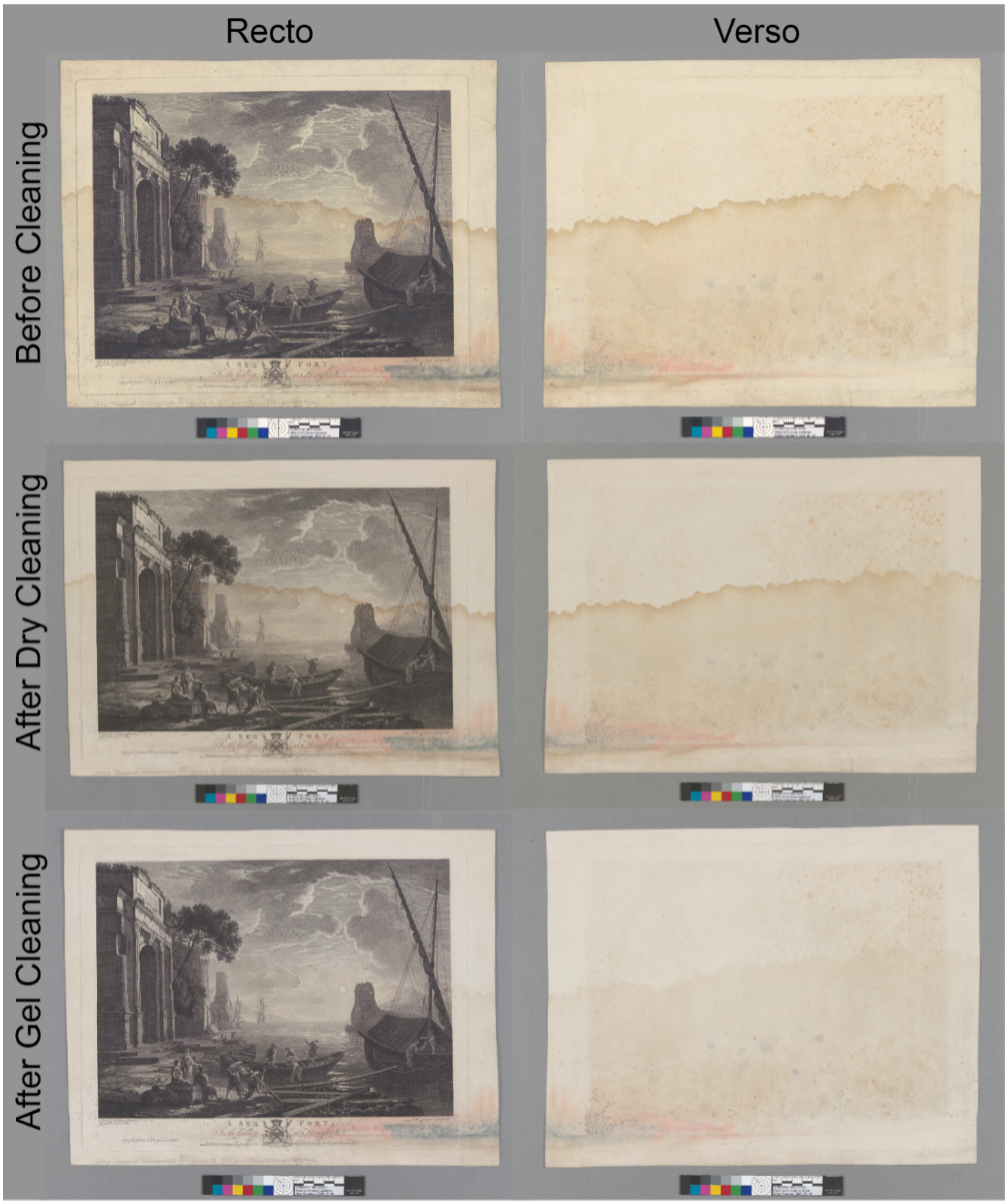

Figure S3: *A Sea Port,* After Claude Lorrain, 1775. National Gallery of Art non-accessioned study collection. Images showing different stages of the overall dry surface cleaning and microcellulose-bulked agarose gel cleaning of the 18th-century print *A Sea Port.*

Table S1: Results of a Tukey test used for statisitical analysis. Sig equals 1 indicates that the difference of the means is significant at the 0.05 level. Sig equals 0 indicates that the difference of the means is not significant at the 0.05 level.

	MeanDiff	SEM	q Value	Prob	Alpha	Sig	LCL	UCL
4% agarose 2% agarose	6.13333	2.76948	3.13194	0.19883	0.05	0	-2.7355	15.00217
2% agarose + 1% SG 2% agarose	14.93333	2.76948	7.6256	0.0029	0.05	1	6.0645	23.80217
2% agarose + 1% SG 4% agarose	8.8	2.76948	4.49366	0.05178	0.05	0	-0.06884	17.66884
2% agarose + 1% MC 2% agarose	15.66667	2.76948	8.00007	0.00213	0.05	1	6.79783	24.5355
2% agarose + 1% MC 4% agarose	9.53333	2.76948	4.86813	0.03573	0.05	1	0.6645	18.40217
2% agarose + 1% MC 2% agarose + 1% SG	0.73333	2.76948	0.37447	0.99298	0.05	0	-8.1355	9.60217

References

[1] K. L. Johnson, *Contact Mechanics*, Cambridge University Press, 1987.

[2] K. L. Johnson, K. Kendall and A. D. Roberts, *Proceedings of the Royal Society of London. Series A, Mathematical and Physical Sciences*, 1971, **324**, 301–313.
